# Automatic identification of posteroanterior cephalometric landmarks using a novel deep learning algorithm: a comparative study with human experts

**DOI:** 10.1038/s41598-023-42870-z

**Published:** 2023-09-19

**Authors:** Hwangyu Lee, Jung Min Cho, Susie Ryu, Seungmin Ryu, Euijune Chang, Young-Soo Jung, Jun-Young Kim

**Affiliations:** 1 Department of Oral and Maxillofacial Surgery, Yonsei University College of Dentistry, 50-1 Yonsei-ro, Seodaemun-gu, Seoul, 03722 South Korea; 2 Research and Development Team, Laon Medi Inc., 404 Park B, 723 Pangyo-ro, Bundang-gu, Seongnam-si, 13511 South Korea; 3 Department of Orthodontics, Yonsei University College of Dentistry, 50-1 Yonsei-ro, Seodaemun-gu, Seoul, 03722 South Korea; 4 Institute for Innovation in Digital Healthcare, Yonsei University, Seoul, 03722 South Korea

**Keywords:** Dentistry, Medical imaging

## Abstract

This study aimed to propose a fully automatic posteroanterior (PA) cephalometric landmark identification model using deep learning algorithms and compare its accuracy and reliability with those of expert human examiners. In total, 1032 PA cephalometric images were used for model training and validation. Two human expert examiners independently and manually identified 19 landmarks on 82 test set images. Similarly, the constructed artificial intelligence (AI) algorithm automatically identified the landmarks on the images. The mean radial error (MRE) and successful detection rate (SDR) were calculated to evaluate the performance of the model. The performance of the model was comparable with that of the examiners. The MRE of the model was 1.87 ± 1.53 mm, and the SDR was 34.7%, 67.5%, and 91.5% within error ranges of < 1.0, < 2.0, and < 4.0 mm, respectively. The sphenoid points and mastoid processes had the lowest MRE and highest SDR in auto-identification; the condyle points had the highest MRE and lowest SDR. Comparable with human examiners, the fully automatic PA cephalometric landmark identification model showed promising accuracy and reliability and can help clinicians perform cephalometric analysis more efficiently while saving time and effort. Future advancements in AI could further improve the model accuracy and efficiency.

## Introduction

Cephalometric analysis is a fundamental diagnostic procedure that utilizes radiological landmarks to measure various linear, angular, and proportional parameters on lateral and posteroanterior (PA) cephalograms^1,2^, which offer valuable information for evaluating craniofacial structures, such as growth assessment, orthodontic treatment planning, orthognathic surgery planning, and treatment outcome assessment^3–5^. Nevertheless, manual diagnostic procedure is a demanding and time-consuming task. Moreover, despite the essential role of landmark identification in cephalometric analysis, intra- and inter-observer variability and a lack of reliability continue to pose a challenge^6,7^. Since the accuracy of landmark identification determines the quality of diagnosis, inaccurate identification of cephalometric landmarks can result in misguided planning of orthodontic therapy and orthognathic surgery.

Therefore, there is a growing need to develop fully automated and reliable cephalometric landmark identification methods that use artificial intelligence (AI) algorithms. Recent advances in AI, particularly in deep learning, have garnered considerable attention for its use in diagnostic imaging, disease classification, and monitoring^8,9^. In orthodontics, deep learning technologies have been utilized for automated cephalometric landmark identification, among other applications^3,10,11^.

Since the 1990s, various studies have proposed fully automated cephalometric landmark identification systems that utilize machine-learning techniques^12,13^. However, their limited accuracy has hindered their success. Recently, deep learning algorithms, such as convolutional neural networks (CNNs), have been used increasingly to detect landmarks on lateral cephalograms automatically^3,14,15^. These studies have reported that deep learning algorithms exhibit high accuracy in detecting landmarks at shorter time, achieving precision levels within the range of 2.0 mm. Additionally, they have achieved successful detection rates (SDR), surpassing 70% and 90% for the respective thresholds of 2 and 3 mm^4,16,19–21^. However, most studies reporting the development and evaluation of automated cephalometric identification algorithms primarily focused on utilizing lateral cephalograms as their main target.

Although lateral cephalometric analysis is valuable in assessing anteroposterior and vertical issues, it has limitations in evaluating skeletal asymmetry and dentofacial structures in the transverse plane. The use of posteroanterior cephalogram holds significant value in the assessment of transverse skeletal and dentoalveolar relationships, enabling the quantification of bilateral structural issues^22–24^. It provides vital diagnostic information that is essential for evaluating patients who present with functional, dentoalveolar, and/or facial asymmetries.

Nevertheless, since the impact of such analyses greatly depends on their accuracy, there are limitations associated with the use of the PA cephalogram owing to errors in landmark identification. There are two primary categories of cephalometric errors: "projection errors," which result from the geometric aspects of the radiographic setup, and "identification errors," which occur owing to the uncertainty in locating specific anatomical landmarks^25,26^. The reliability and reproducibility of identified landmarks are influenced by various factors, including image density, image sharpness, anatomical complexity, and superimposition of anatomical structures^27–29^. Consequently, the identification errors on PA cephalograms were higher than those of lateral cephalometric analysis owing to their nature, which involves more superimposition of anatomical structures and variations in head positioning^30^. Furthermore, the experience and predisposition of the examiner play a significant role, asrdeeper understanding of anatomy and familiarity with radiographic images can help reduce intra- and inter-examiner errors^22,31^.

Considering the limitations and potential errors associated with manual landmark identification in PA cephalograms, there is a growing need for automatic identification methods. The development of automatic PA cephalogram identification systems can greatly enhance the accuracy and reliability of the analysis, minimizing the risk of errors caused by human factors. Moreover, the implementation of automatic PA cephalogram identification would not only expedite the analysis, but also contribute to enhanced diagnostic capabilities and more effective treatment planning for patients presenting with functional, dentoalveolar, and/or facial asymmetries. Therefore, this study aimed to develop a fully automatic PA cephalometric landmark identification model using a deep learning algorithm and compare its accuracy and reliability with the assessments made by expert human examiners.

## Results

Table 1 presents the mean radial error (MRE) and intra-class correlation coefficient (ICC) values for each of the 19 landmarks detected by the human examiners that were defined as the gold standard points. The average MRE for all landmarks was 1.68 ± 1.85 mm. The ICC values for all landmarks were above 0.7, except for the y-coordinate of the upper dental midline (U1M), indicating high reliability between the two examiners. The lower ICC value for the y-coordinate of the U1M may be attributed to the detection tendency of the two human examiners, which is relatively less relevant to transverse evaluation.

**Table 1:** Mean radial error (MRE) and inter-examiner reliability of two human examiners.

Landmarks	Mean radial error (mm)		Interclass correlation coefficient	
	Mean	SD	x-coordinate	y-coordinate
Cg	3.53	2.65	0.983	0.783
SphR	0.94	0.75	0.989	0.986
SphL	1.17	0.92	0.982	0.979
ConR	3.61	2.34	0.886	0.771
ConL	3.95	2.56	0.859	0.725
MstR	0.85	0.60	0.991	0.988
MstL	0.81	0.75	0.991	0.987
ANS	0.89	0.68	0.994	0.986
JugR	1.42	1.42	0.987	0.953
JugL	1.11	1.16	0.993	0.968
GoR	1.68	1.73	0.983	0.952
GoL	1.60	1.37	0.980	0.969
Me	1.46	1.23	0.957	0.995
U1M	2.32	11.21	0.710	0.531
U6MCR	1.61	1.08	0.966	0.970
U6MCL	1.41	1.13	0.973	0.977
L1M	1.13	1.35	0.958	0.993
L6MCR	1.13	1.04	0.973	0.987
L6MCL	1.21	1.20	0.971	0.985
Average	1.68	1.85	–	–

**Figure 1:**
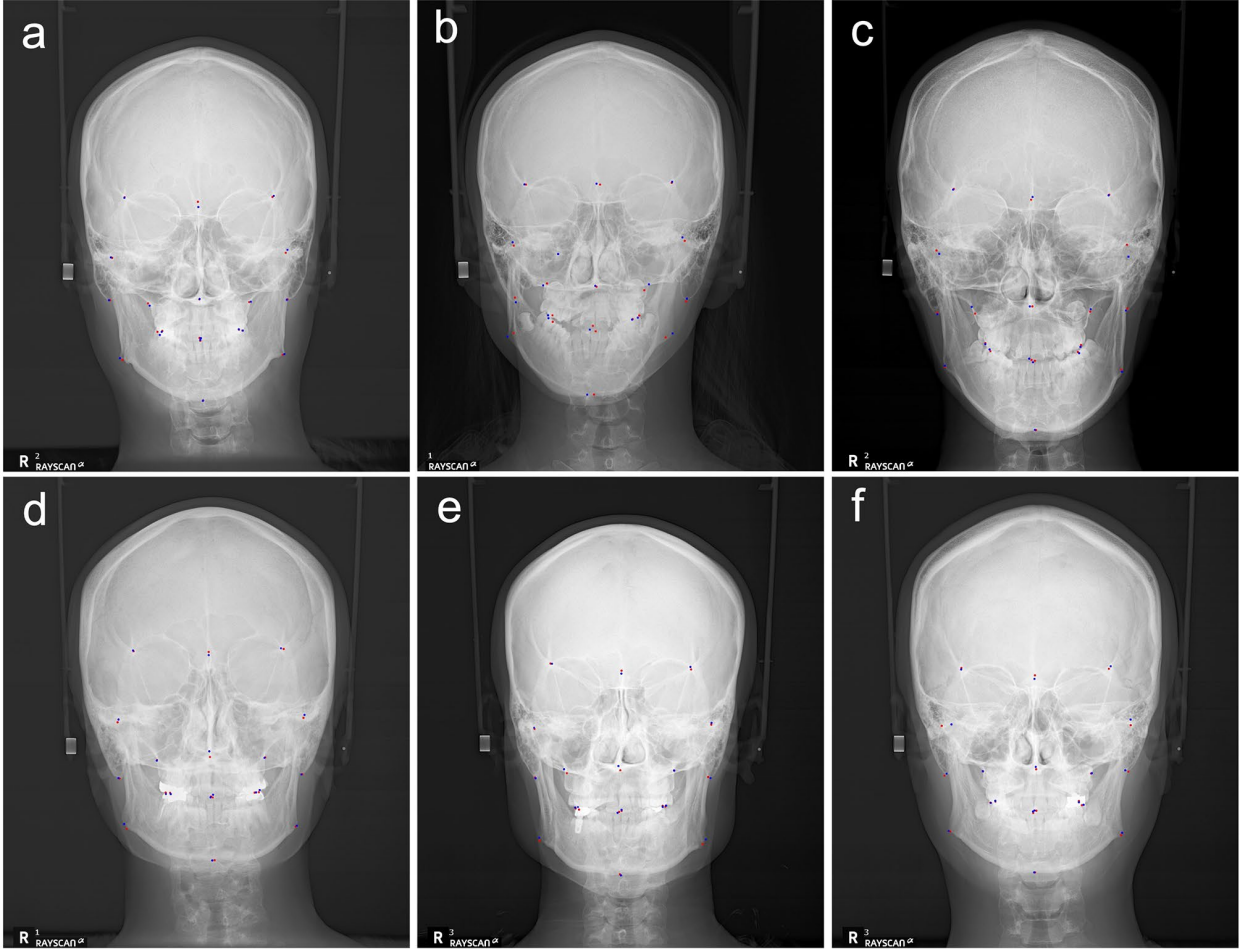
Examples of superimposed gold standard (blue dot) and auto-identified (red dot) landmarks on PA cephalometric image (**a**–**f**). The best result was obtained with an average MRE of 1.06 mm (**a**), and the worst result with an average MRE of 4.02 mm (**b**).

Examples of the 19 landmarks were visually represented on a PA cephalometric image to facilitate comparison between the landmarks detected by the human examiners (gold standard) and those by the automatic identification algorithm (Fig. 1).

Table 2 presents the MRE and standard deviation (SD) for the 19 landmarks identified by the human examiners (the gold standard) and the automatic identification algorithm. The landmarks with the lowest MRE were SphR (1.12 mm), SphL (1.10 mm), and MstR (1.10 mm), whereas those with the highest MRE were ConR (3.47 mm) and ConL (3.16 mm). The average MRE was 1.87 ± 1.53 mm.

**Table 2:** Mean radial error (MRE) between gold standard and auto-identification using the AI algorithm.

Landmarks	Mean radial error (mm)	
	Mean	SD
Cg	2.57	1.63
SphR	1.12	0.97
SphL	1.10	0.67
ConR	3.47	2.09
ConL	3.16	1.88
MstR	1.10	1.16
MstL	1.95	1.28
ANS	1.83	1.29
JugR	1.91	1.59
JugL	1.70	1.24
GoR	1.70	1.34
GoL	1.49	1.20
Me	2.17	1.77
U1M	2.19	5.54
U6MCR	1.55	0.99
U6MCL	1.43	0.78
L1M	2.50	1.83
L6MCR	1.33	0.98
L6MCL	1.18	0.76
Average	1.87	1.53

Table 3 shows the SDR of the landmarks within the error ranges of < 1.0, < 2.0, and < 4.0 mm. The average SDR was 34.7%, 67.5%, and 91.5% within the ranges of 1.0, 2.0, and 4.0 mm, respectively. The automatic identification algorithm showed a high accuracy (> 80%) within a range of 2.0 mm for SphR and SphL (89.0%), MstR and L6MCL (87.8%), and U6MCL and L6MCR (81.7%). In contrast, ConR (25.6%) and ConL (32.9%) had the lowest SDR values.

**Table 3:** Success detection rate (SDR) of auto-identification using the AI algorithm.

Landmarks	Success detection rate (%)		
	< 1.0 (mm)	< 2.0 (mm)	< 4.0 (mm)
Cg	14.6	45.1	79.3
SphR	53.7	89.0	98.8
SphL	51.2	89.0	100.0
ConR	6.1	25.6	73.2
ConL	12.2	32.9	69.5
MstR	65.9	87.8	98.8
MstL	19.5	57.3	93.9
ANS	29.3	61.0	93.9
JugR	36.6	61.0	92.7
JugL	29.3	68.3	91.5
GoR	39.0	70.7	93.9
GoL	43.9	75.6	97.6
Me	26.8	67.1	85.4
U1M	50.0	72.0	92.7
U6MCR	35.4	74.4	97.6
U6MCL	30.5	81.7	100.0
L1M	20.7	53.7	81.7
L6MCR	46.3	81.7	98.8
L6MCL	47.6	87.8	100.0
Average	34.7	67.5	91.5

**Figure 2:**
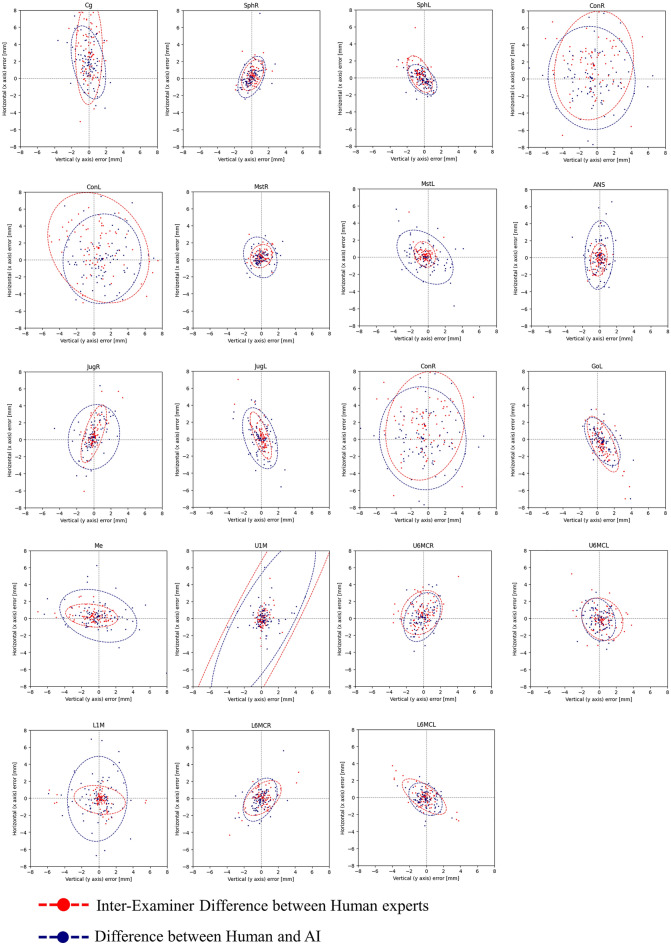
Scatter plots with 95% confidence ellipses for the landmark detection errors of the human examiners and automatic identification.

The horizontal and vertical error patterns between the two human examiners and the automatic identification of each landmark are illustrated in Fig. 2.

## Discussion

This study proposed a fully automated deep learning model for identifying PA cephalometric landmarks and compared its accuracy with that of two expert human examiners.

Numerous studies have introduced algorithms for the automated identification of lateral cephalograms as well as methods for evaluating their accuracy^3,10,11^. Conversely. A recent systematic review and meta-analysis reported AI agreement rates of 79% and 90% for the thresholds of 2 and 3 mm, respectively, with a mean divergence of 2.05 compared to manual landmarking^19^. Another study showed that most studies did not exceed a 2-mm prediction error threshold in mean and that the mean proportion of landmarks detected within this 2-mm threshold was 80%^20^.

However, only a limited number of studies have used deep learning algorithms to automate identification in PA cephalograms^18^. Although lateral cephalometric analysis is a basic tool for diagnosis in orthodontics, PA cephalometric analysis is essential for evaluating skeletal asymmetry and providing dentofacial structural information in the transverse plane^22–24^. Owing to its anatomical orientation, PA cephalometry inevitably causes greater overlap and superimposition of the skeletal structures and dentition than lateral cephalometry. These overlapped images reduce the accuracy and reliability of landmark identification, resulting in identification that highly depends on the examiner's experience and subjectivity^22,31–33^.

The present study aimed to develop a fully automatic PA cephalometric landmark identification system using a two-step landmark detection framework. In the first step, ResNet 18 was used to detect the region of interest by roughly extracting 19 landmark positions. Random augmentation and loss functions were used to improve performance. In the second step, ResNet 50 architecture was used to extract fine points from the cropped image. Then, CLAHE was applied to the cropped images for clear visualization of the bone, soft tissue, and the background regions.

The inference time and accuracy according to number of ResNet layers during the coarse training step are listed on Table 4. Deeper networks can extract more complex and abstract features. However, such complex feature extraction comes at the cost of increased computational burden, resulting in longer inference times. Similarly, as shown in the Table 4, as the layer depth increased, the computing time also increased. Although ResNet34 demonstrated approximately a 19% improvement in average accuracy for landmark detection compared to ResNet 18, its inference time was approximately 40% slower. Therefore, we opted to use ResNet18 for the coarse training stage.

**Table 4:** Inference time and accuracy according to the number of ResNet layers.

Model	Average computing time (s)	Average radial error (mm)
ResNet 18	0.005573844	4.262201158
ResNet 34	0.009241667	3.583836895
ResNet 50	0.012178767	7.023593526
ResNet 152	0.034862056	7.182261526

To evaluate the performance of the proposed model, manual identification of the 19 landmarks was performed independently by two human examiners on 1032 images used for model training and validation. Furthermore, the landmarks were identified manually by two expert human examiners and automatically by the developed AI algorithm on 82 test set images. The MRE and SDR between gold standard and auto detected point were calculated from the results of the test sets.

Although every cephalometric landmark has its own definition, its identification is subjective and dependent on the experience and judgment of the examiner^22,31^. Even highly educated experts may hold different opinions regarding the ideal location, indicating the absence of an 'absolute gold standard' reference point. To address this limitation, we employed a method where the arithmetical mean point for each landmark was calculated by averaging the X and Y coordinates detected by two human expert examiners. The inter-examiner reliability was previously established using an assessment of the ICC. Although this mean point may not precisely align with the specific definition of a particular landmark, it can be regarded the closest approximation to the gold standard point.

The SphR, SphL, and MstR had the lowest MRE, whereas the condyle points had the highest MRE. The average MRE value was 1.87 mm, which falls within the clinically acceptable range of previous studies that considered an MRE of up to 2.0 mm as acceptable^3,10,11,34^. In terms of the SDR, auto-identification demonstrated a high accuracy at the sphenoid points and the mastoid process across all error ranges. Conversely, the lowest SDR was observed at the condyle points.

The tendency of these results could be related to the degree of overlap of the anatomical structures. Auto-identification showed low MRE and high SDR values for the sphenoid points and mastoid processes, respectively, which have relatively little anatomical overlap. Conversely, auto-identification exhibited high MRE and low SDR values for the condyle points, which overlap with the maxilla and zygomatic arch, respectively, consistent with the inter-examiner evaluation. These results align with those of prior studies on auto-identification on PA cephalograms, which reported relatively significant errors in identifying the landmarks located within the frontozygomatic suture, zygomatic process, and condyles owing to an overlap of the anatomical structures^17,22,35^.

The precision of auto-identification of the landmarks located on the teeth, which was anticipated to have low accuracy owing to the overlap of adjacent teeth and the presence of orthodontic brackets and prosthesis, produced favorable results, with an MRE within 2.0 mm. This finding suggest that auto-identification can be utilized for precise evaluation of asymmetry using the dentition.

The scatterplots of the landmark detection errors showed a characteristic distribution within the co-ordinate system, as shown in Fig. 2. For instance, Menton (Me) distinctively oriented horizontally, whereas Crista galli(Cg) oriented more in a vertical direction, which reflects the tendency of detection errors of each landmarks and corresponding to the differences observed between the two examiners.

In conclusion, this study presented a novel approach for the fully automatic identification of PA cephalometric landmarks using a deep learning algorithm. The accuracy and reliability of the proposed model were evaluated by comparison with those of expert human examiners. Our results showed that the accuracy and reliability of the constructed AI model are comparable to those of human experts. These findings suggest that with advances in AI, automatic PA cephalometric landmark identification can significantly improve the efficiency and accuracy of cephalometric analysis while reducing the time and effort required.

## Methods

This study was approved by the Ethics Review Board of Yonsei University Dental Hospital Institutional Review Board (approval number 2–2020-0005) and passed the exemption review of informed consent on the use of patients’ cephalometric data. The requirement for written or verbal informed consent was waived owing to the non-interventional retrospective study design, and all cephalometric images were anonymized to ensure confidentiality. This study was performed in accordance with the Declaration of Helsinki.

The inclusion criteria were as follows: (1) patients aged between 18 and 39 years, with permanent dentition and complete facial growth, and (2) those who underwent orthodontic therapy or orthognathic surgery between 2015 and 2021. The exclusion criteria were as follows: (1) partial or total edentulism, and (2) a history of dentofacial trauma, craniofacial syndromes, or systemic diseases. Thus, 1114 PA cephalometric images taken before treatment from the participants who met the inclusion criteria and were included in this study.

The PA cephalograms used in this study were acquired using a Rayscan machine (Ray Co. Ltd., Hwaseong, Korea) and collected from the picture archiving and communication system of the Yonsei University Dental Hospital as JPEG files. The images had a resolution of 1930 × 2238 pixels and a pixel spacing of 0.13 mm. Each pixel was represented by a single grayscale channel with values ranging from 0–255.

The 1114 PA cephalometric images included in the study were randomly divided into three sets: 803 images for training purposes, 229 for validation, and 82 for testing. The training and validation sets were used exclusively during the model training phase, whereas the test set was used solely to evaluate the reliability of the human examiners and the accuracy of the auto-identification model.

**Figure 3:**
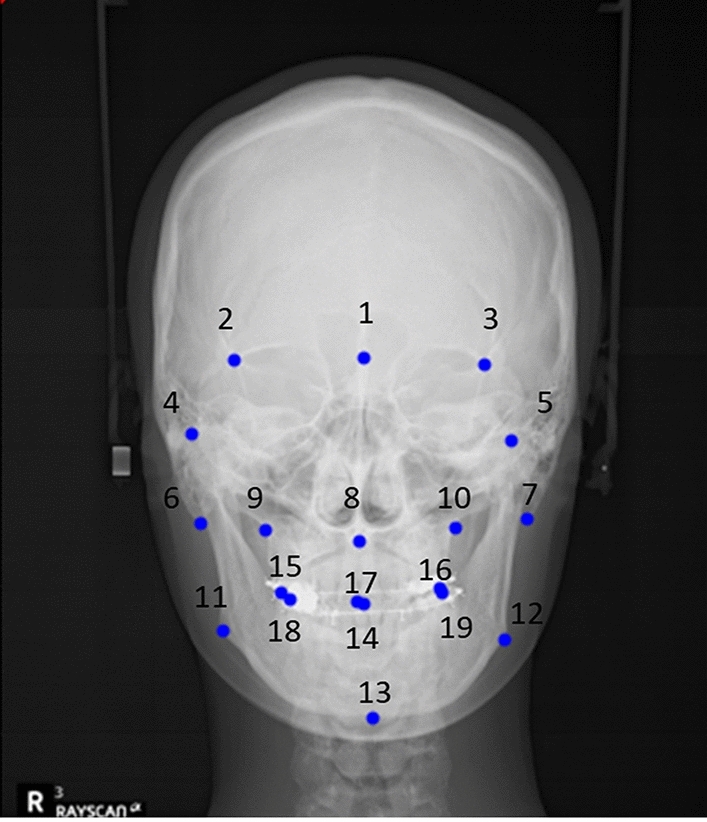
Landmarks on the PA cephalometric radiograph.

A total of 19 clinically important PA cephalometric landmarks used in routine dentofacial diagnosis were selected. Table 5 and Fig. 3 describe their definitions and positions. Two expert human examiners, an oral and maxillofacial surgeon with over 10 years of clinical experience in dentofacial deformity and an orthodontic specialist with 5 years of orthodontic training, independently and manually identified the landmarks on the 1032 images used for model training and validation to obtain the ground truth.

**Table 5:** Definition of landmarks.

No	Landmarks	Definition
1	Crista galli (Cg)	The most superior and anterior points on the median ridge of the bone that projects upward from the cribriform plate of the ethmoid bone
2	Sphenoid point right (SphR)	The right intersection of sphenoid bone greater and lesser wing
3	Sphenoid point left (SphL)	The left intersection of sphenoid bone greater and lesser wing
4	Condyle point right (ConR)	The most superior and the middle point on the contour of the right condyle head
5	Condyle point left (ConL)	The most superior and the middle point on the contour of the left condyle head
6	Mastoid process right (MstR)	The most inferior point of the right mastoid process
7	Mastoid process left (MstL)	The most inferior point of the left mastoid process
8	Anterior nasal spine (ANS)	The center of the intersection of the nasal septum and the palate
9	Jugal point right (JugR)	The right intersection of the tuberosity of maxilla and zygomatic buttress
10	Jugal point left (JugL)	The left intersection of the tuberosity of maxilla and zygomatic buttress
11	Gonial point right (GoR)	The most posterior inferior point of the right mandibular angle
12	Gonial point left (GoL)	The most posterior inferior point of the left mandibular angle
13	Menton (Me)	The most inferior point of the symphysis of the mandible
14	Upper dental midline (U1M)	The dental midline point of incisal edge of the maxillary central incisor
15	Upper first molar cusp right (U6MCR)	The most lateral cusp point of the right maxillary first molar crown
16	Upper first molar cusp Left (U6MCL)	The most lateral cusp point of the left maxillary first molar crown
17	Lower dental midline (L1M)	The dental midline point of the incisal edge of the mandibular central incisor
18	Lower first molar cusp right (L6MCR)	The most lateral cusp point of the right mandibular first molar crown
19	Lower first molar cusp left (L6MCL)	The most lateral cusp point of the left mandibular first molar crown

During model training, the large size of the original image facilitates the creation of more feature maps for learning; however, it is also associated with the disadvantages of GPU memory allocation limitation and long computing time. Therefore, in the first step, the image was resized to 964 × 1119 pixels, which was ¼^th^ of the original size. It is important to retain the features of the widest possible area to extract the approximate coordinates of the 19 landmarks. Thus, the x- and y-coordinates of the 19 landmarks were extracted by locating the center of mass of each labeling point, which enabled the construction of a coordinate landmark detection model.

**Figure 4:**
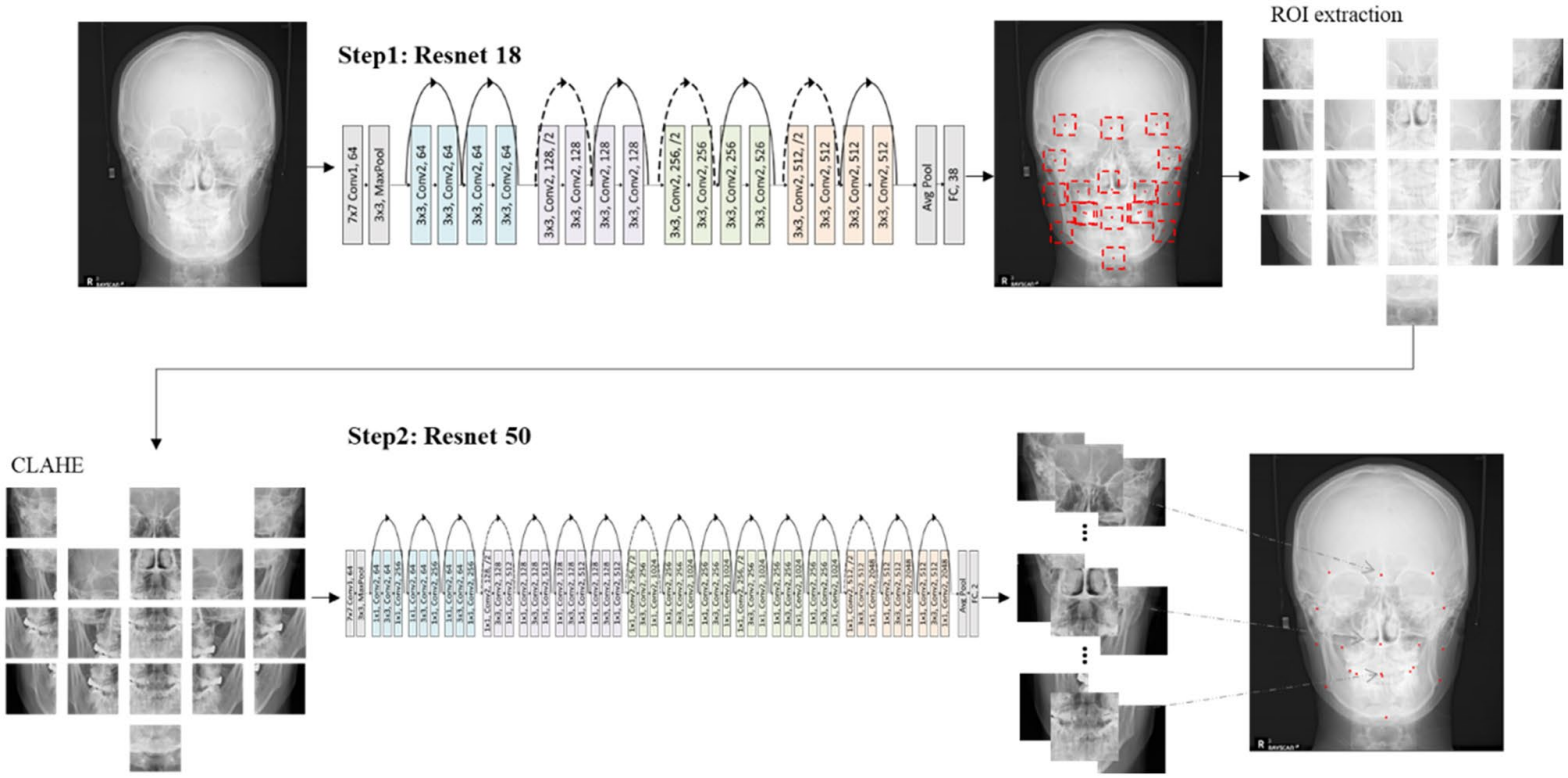
Schematic design of the two-step landmark detection framework. (Conv: convolution, FC: fully connected, ROI: region of interest, CLAHE: Contrast Limited Adaptive Histogram Equalization).

The landmark detection framework operates through a two-step process, as shown in Fig. 4. The 19 landmark positions were coarsely extracted, and the image was cropped to a certain size based on these rough positions. Subsequently, the fine points were extracted from the cropped images. The adoption of this two-step framework facilitated efficient learning and high accuracy.

ResNet 18 was used as the initial step to preserve the original features and expedite the learning process while minimizing the computational complexity. ResNet 18 is a model that can solve the gradient vanishing problem as the layer deepens through residual learning using skip connection, and it is widely used in facial landmark detection tasks. ResNet 18 consists of 17 convolution layers and a fully connected layer at the end. However, the first convolution layer was limited to a 7 × 7 kernel and max pooling to minimize the input size, whereas all subsequent layers were implemented using a 3 × 3 kernel convolution layer. The final fully connected layer comprised 38 output features, thereby enabling the derivation of the x- and y-coordinates of the 19 landmarks. Residual shortcut connections were introduced between the two convolution layers to optimize the learning process, as shown in Fig. 4, where the solid line represents the input and output having the same dimension, and the dotted line represents an increase in the dimension with zero padding and a stride of 2.

**Figure 5:**
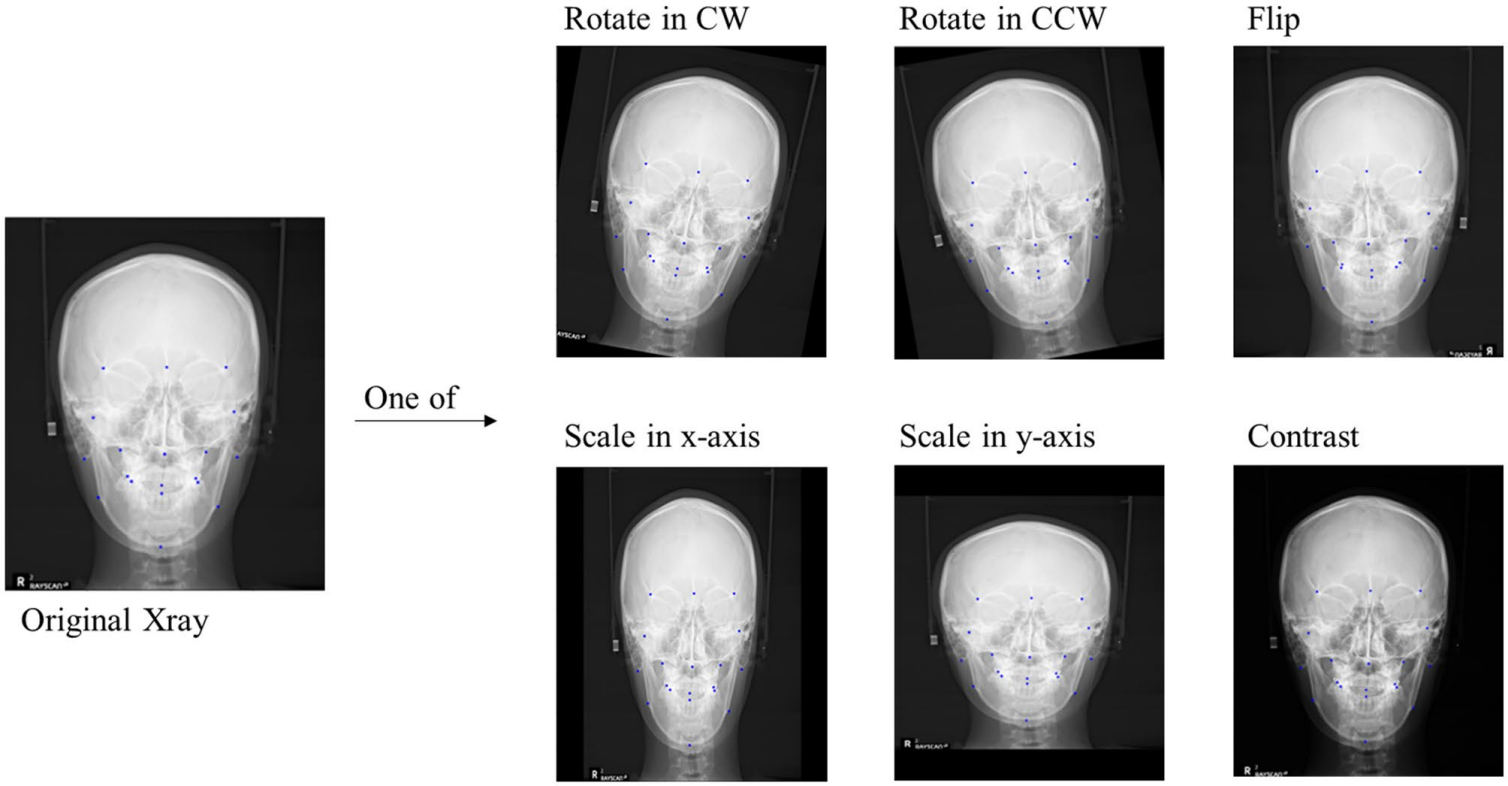
Description of the augmentation policy (CW: clockwise, CCW: counterclockwise).

An augmentation strategy randomly selected from a list of methods, including rotation, scale, flip, and contrast, was applied to account for patients with tilted heads and asymmetric X-rays, as shown in Fig. 5. Wing loss was utilized as the loss function in the first step, which helped reduce an excessive focus on outliers to find the approximate landmark positions^36,37^. Wing loss is more resistant to the impact of outliers than the mean squared error (MSE) loss function.

In the second step, the original image was cropped to a size of 400 × 400 pixels and centered around the 19 landmark positions obtained in the first step. Subsequently, contrast-limited adaptive histogram equalization (CLAHE) was applied to the cropped images. CLAHE is a histogram-flattening method that enhances the contrast of the radiographs, thereby enabling clear visualization of the bone, soft tissue, and background regions^38^. The application of CLAHE is known to enhance image quality and has gained widespread usage in deep learning model studies that utilize medical images^39,40^. ResNet 50 architecture was used in this step. It is similar to ResNet 18 but with deeper networks, and it comprises 49 convolution layers and a fully connected layer at the end. The final fully connected layer was designed with two output features to derive the x- and y-coordinates of one landmark. To optimize learning, residual shortcut connections were applied to three convolution layers with kernel sizes of 1 × 1, 3 × 3, and 1 × 1. The 1 × 1 convolution layers were responsible for dimensionality reduction and restoration, whereas the 3 × 3 layer functioned as a bottleneck with smaller input/output dimensions. MSE loss was utilized as the loss function for the second step.

The two-step models were initialized with a learning rate of 0.01 during model training, which was then decayed by a factor of 0.5 every 30 epochs. An Adam optimizer with a batch size of 64 was used for 400 epochs. All procedures were conducted using the PyTorch framework running on an NVIDIA Quadro RTX8000 GPU.

Two expert examiners specializing in oral and maxillofacial surgery and orthodontics manually identified 19 cephalometric landmarks on 82 images that constituted the test set. The MRE and SD were calculated to evaluate the inter-examiner reliability, and the ICC was computed to assess the degree of reliability between the two human experts. The mean values of the x- and y-coordinates determined by the two examiners were used as the gold standard for subsequent analysis.

Automatic detection of 82 test set images was completed using the constructed AI algorithm, and the MRE and SDR with error ranges of < 1.0, < 2.0, and < 4.0 mm for all landmarks were calculated to evaluate the performance of the proposed model. All calculations were performed in Microsoft Excel using the following formulae:

$${\text{Radial}}\,{\text{error}}\,({\text{R}}) = \sqrt {\Delta x^{2} + \Delta y^{2} } \quad ({\text{mm}})$$

$${\text{MRE}} = \frac{{\mathop \sum \nolimits_{i = 1}^{N} R_{i} }}{N} \quad ({\text{mm}})$$

$${\text{SDR }} = \frac{{{\text{Number}}\,{\text{of}}\,{\text{accurate}}\,{\text{identifications}}}}{{{\text{Number}}\,{\text{of}}\,{\text{total}}\,{\text{identifications}}}} \times100\%$$

## Data Availability

The datasets used and/or analyzed during the current study are available from the corresponding author on reasonable request.
